# 4281 Developing a predictive tool to detect peripheral artery disease (PAD): Examining patient-reported symptoms in ischemic versus non-ischemic conditions (PREDICT PAD)

**DOI:** 10.1017/cts.2020.120

**Published:** 2020-07-29

**Authors:** Rebecca Brown, Erica Schorr, Diane Treat-Jacobson

**Affiliations:** 1 University of Minnesota; 2 University of Minnesota School of Nursing

## Abstract

OBJECTIVES/GOALS: Objectives: The study goal is to identify discriminating symptom characteristics of PAD versus non-ischemic conditions to improve recognition. Just as nausea, back, and jaw pain were once thought to be unrelated to myocardial infarction and coronary artery disease, patient-reported symptoms of PAD are frequently overlooked as being a sign of PAD. METHODS/STUDY POPULATION: Methods: Using a prospective de novo population-based cross-sectional design we will link symptom descriptors to PAD disease status using diagnostic testing in individuals who report lower extremity or buttock symptoms (n = 100). Symptom descriptors will be obtained via questionnaires and structured interviews will be completed pre and post physical function tests. Using near infrared spectroscopy, we will measure calf muscle tissue oxygenation levels to further differentiate ischemic vs. non-ischemic symptoms during exercise. The primary outcome will be the diagnostic accuracy of patient-reported symptoms which discriminate between PAD and non-PAD conditions. Positive predictive value and accuracy will be calculated using receiver operating characteristic (ROC) curve and chi-square analysis. RESULTS/ANTICIPATED RESULTS: Results: Previous studies from which symptom descriptors have been obtained were from patients with known PAD, of which 85-88% of participants were male.^1–2^ Seventy-six percent of this sample thus far is female. Nationally, PAD prevalence is 20% in those over the age of 70 years, however 58% of our study participants tested positive for PAD (via ankle brachial index test).^3^ The most commonly reported symptoms of PAD are “numbness” and “aching” vs. those without PAD most commonly reporting “cramping”. These results trend against our current understanding of PAD symptomatology, which is that cramping is the cardinal symptom of PAD.^4^ Preliminary analysis suggests that balance is a sensitive and specific predictor of PAD. Recruitment is ongoing, therefore results are preliminary. DISCUSSION/SIGNIFICANCE OF IMPACT: Translation of the results will impact primary care and community health. Improved disease detection will position providers to refer patients to exercise therapy before symptoms become disabling. Understanding the diagnostic accuracy of symptoms prepares us to apply novel techniques, such as statistical modeling, to systematically predict PAD.

